# Editorial: The impact of metabolic disorders on female reproductive health

**DOI:** 10.3389/frph.2024.1436451

**Published:** 2025-01-16

**Authors:** Lokesh Kumar, Vineet K. Maurya, Manasi Kamat

**Affiliations:** ^1^ Genus Plc, ABS Global, Windsor, WI, United States; ^2^ Department of Molecular and Cellular Biology, Baylor College of Medicine, Houston, TX, United States; ^3^ Amazon, Milpitas, CA, United States

**Keywords:** metabolic disorder, female reproductive health, preeclampsia, female infertility, diabetes mellitus

**Editorial on the Research Topic**
The impact of metabolic disorders on female reproductive health

Metabolic syndrome is a multifaceted condition characterized by a cluster of endocrine and inflammatory pathologies, including obesity, hyperglycemia, hypertension, and insulin resistance. The global prevalence of metabolic-associated disorders has escalated in recent decades, significantly impacting gynecological health. These disorders disrupt hormonal regulation, ovarian function, fertilization, and pregnancy, leading to various complications such as ectopic pregnancy, pelvic inflammatory disease, polycystic ovary syndrome (PCOS), anovulation, menorrhagia, miscarriage, recurrent pregnancy loss (RPL), gestational diabetes mellitus (GDM), and preterm birth. This underscores an urgent need for effective, evidence-based, and patient-centered therapeutic interventions to address these gynecological challenges. This research topic aims to unravel the metabolic alterations that influence female reproductive health and to identify innovative therapeutic approaches.

Zhang et al. investigated the relationship between metabolic alterations and hormonal profiles, highlighting their role in the onset of reproductive disorders, particularly PCOS. The study assessed hormonal markers including follicle-stimulating hormone, luteinizing hormone, estrogen, prolactin, total testosterone, and androstenedione in 160 women with PCOS compared with 139 age-matched controls. Their findings reveal that women with PCOS, both obese and non-obese, exhibit more severe insulin resistance and sex hormone imbalances than their counterparts without PCOS. The study also established a correlation between body fat, glucose metabolism, and hormonal profiles in women with PCOS, offering critical insight into the metabolic underpinnings of this disorder.

In a comprehensive review, Malik et al. summarized existing research on PCOS, its underlying causes, adverse impacts on reproductive physiology, and potential natural plant-based therapeutic interventions. The review detailed preclinical and clinical studies of phytochemicals, providing insight into their effective dosages, study designs, duration, and mechanisms of action. The authors explained that several phytochemicals, such as curcumin, berberine, resveratrol, quercetin, hesperidin, and genistein, have shown promise in preclinical studies for the treatment of PCOS. Compounds like *Vitex agnus-castus*, *Cinnamomum cassia*, pomegranate juice, spearmint tea, *Nigella sativa*, and *Tribulus terrestris* have demonstrated efficacy in managing PCOS in clinical patients. These natural compounds offer potential alternatives or may improve treatment outcomes when used alongside conventional therapies. This work underscores the potential of phytochemical-based treatments as safer, low-toxicity alternatives for PCOS management, paving the way for innovative therapeutic strategies.

The investigation of novel biomarkers is another critical area for advancing diagnostic tools for female reproductive disorders. Marek-Iannucci et al. explored the potential of natriuretic peptides as biomarkers for predicting preeclampsia (PE), a life-threatening condition that is particularly prevalent in low-income regions. Their study analyzed four cases of pregnant women with severe PE, all of whom exhibited elevated levels of brain natriuretic peptide (BNP) in their urine. The authors proposed BNP as a promising biomarker for identifying PE, especially in women with chronic hypertension, facilitating earlier and more effective intervention.

The past decade has also witnessed growing interest in benign uterine conditions, such as adenomyosis (ADS), and their association with female infertility. However, the underlying pathogenic mechanisms of ADS remain inadequately understood. Wang and Duan examined the link between ADS and infertility, emphasizing the role of the myometrium's junctional zone in maintaining uterine fertility. Altered contractility within this zone can significantly impair reproductive outcomes. Leveraging advanced diagnostic tools such as MRI, ultrasound, and imaging, the authors highlighted the intricate dynamics of the junctional zone and reviewed various therapeutic options for addressing ADS-related infertility.

This editorial highlights the critical intersections of metabolic alterations, hormonal imbalances, and gynecological health. From novel phytochemical treatments for PCOS to emerging biomarkers for preeclampsia and insights into adenomyosis-related infertility, these studies collectively emphasize the importance of innovative and multidisciplinary approaches to advancing women's reproductive health.

Any metabolic disturbances can significantly disrupt women's physiology, often resulting in reproductive dysfunction. The study by Qin et al.'s investigation examines the negative impact of type 2 diabetes mellitus on female reproductive outcomes during assisted reproductive techniques. The study analyzed 265 infertile female patients undergoing assisted reproduction and found that those with type 2 diabetes mellitus had lower levels of anti-Müllerian hormone (AMH) and worse pregnancy outcomes than those without diabetes. This underscores the diminished ovarian reserve in diabetic women compared to their nondiabetic counterparts.

This special issue encompasses a diverse array of articles that shed light on the interplay between metabolic disorders and pregnancy, fundamentally reshaping our understanding of metabolic disorders associated with infertility and how they affect pregnancy in humans.

